# Vagus nerve stimulation: anti-inflammatory effects in epilepsy

**DOI:** 10.3389/fnhum.2026.1797556

**Published:** 2026-04-22

**Authors:** Veronika Abzalova, Sholpan Kauynbekova, Gabit Makhambayev, Aleksandr Dmitriev, Berik Tuleubayev, Adil Koshkinbayev, Arsen Dauletov

**Affiliations:** 1 Department of Surgical Diseases, Karaganda Medical University, Karaganda, Kazakhstan; 2 Multidisciplinary Hospital Named after Professor Kh.Zh. Makazhanov, Karaganda, Kazakhstan; 3 Center for New Medical Technologies, Novosibirsk, Russia

**Keywords:** biomarkers, drug-resistant epilepsy, neuroinflammation, neuromodulation, seizures

## Abstract

**Introduction:**

Vagus Nerve Stimulation (VNS) is an established standard of care for drug-resistant epilepsy; however, the biological mechanisms underlying its cumulative therapeutic effect remain incompletely understood. This study aims to evaluate the monitoring value of neuroinflammation and neurodegeneration biomarkers to objectify the therapeutic response.

**Methods:**

In this prospective longitudinal study, we evaluated 40 pediatric patients (20 receiving active VNS therapy and 20 age-matched controls). Plasma levels of UCHL-1, HMGB1, and NSE were investigated as potential indicators of blood–brain barrier status, neuroinflammation, and metabolic stress, respectively. Measurements were performed at baseline and after 3, 6, and 12 months of treatment. Clinical efficacy was defined by the reduction in seizure frequency.

**Results:**

The primary focus of the analysis was on the active VNS therapy group, where median seizure frequency decreased by 44.4% (*p* < 0.001) by month 12. For comparison, the clinical profile and biomarker levels in the control cohort did not change significantly throughout the year (all *p* > 0.05), confirming the specificity of neurostimulation effects. In the active group, biomarker profiling revealed a temporal dissociation in the biological response. UCH-L1 levels demonstrated a significant decrease by month 6 (*p* = 0.009), potentially reflecting an early functional stabilization of the blood–brain barrier. In contrast, HMGB1 concentrations showed a significant reduction only by month 12 (*p* < 0.001), which strongly correlated with clinical improvement (*r* = 0.63). Notably, NSE levels exhibited no significant longitudinal changes during the observation period.

**Conclusion:**

VNS efficacy appears to be associated with a complex, multi-phasic biological response. The temporal dynamics of peripheral biomarkers may reflect a potential early stabilization of the blood–brain barrier, followed by delayed systemic immunomodulation. While blood-based analysis precludes direct conclusions regarding central neuroinflammation, the delayed reduction of circulating inflammatory signals points to a systemic anti-inflammatory effect that likely contributes to the cumulative therapeutic benefits. Thus, dynamic assessment of these accessible neuroimmune proteins provides an objective systemic correlate of clinical improvement. This biomarker panel may serve as a valuable supportive tool for monitoring VNS therapeutic response and guiding personalized neuromodulation parameters.

## 1 Introduction

Drug-resistant epilepsy, affecting up to 30% of the epileptic population, remains a critical challenge in modern neurology. This condition is associated with a substantial disease burden, particularly within the pediatric population, where persistent seizure activity exerts a devastating impact on neurodevelopment, cognitive function, and social adaptation. For patients who are not candidates for resective surgery due to multifocal lesions, the localization of foci within eloquent cortical areas, or the complexity of the epileptogenic network, VNS represents the “gold standard” of neuromodulation and palliative care. However, the clinical response to VNS exhibits significant variability, ranging from complete seizure freedom to a lack of therapeutic effect (Löscher et al., 2020; Nickels et al., 2016).

Currently, therapeutic efficacy is frequently assessed retrospectively, relying heavily on subjective clinical observation and seizure diaries. This necessitates the search for objective biomarkers capable of verifying the baseline level of neuroinflammation and confirming the targeted biological impact of VNS over time. The dynamic monitoring of such biomarkers could provide a reliable objective correlate of the clinical response, thereby unlocking the potential for timely adjustment of neuromodulation parameters and personalized therapy management (Englot et al., 2011; Vezzani et al., 2011; Vezzani and Friedman, 2011).

Over the past decades, the paradigm of epilepsy pathogenesis has shifted toward recognizing the critical role of neuroinflammation (Andersson and Tracey, 2012). Characterized by microglial and astrocytic activation, blood–brain barrier (BBB) dysfunction, and the release of pro-inflammatory cytokines, this process establishes a vicious cycle that perpetuates neuronal hyperexcitability (Rana and Musto, 2018).

The fundamental mechanisms underlying the barrier-stabilizing effects of VNS have been elucidated primarily through experimental animal models. Preclinical studies have convincingly demonstrated the robust neuroprotective properties of VNS. Specifically, it reduces cerebrovascular permeability, prevents the degradation of tight junction proteins, and downregulates the expression of matrix metalloproteinases in reactive perivascular astrocytes (Lopez et al., 2012; Chen et al., 2018; Yang et al., 2018). This effect is hypothesized to be mediated by the local release of acetylcholine and norepinephrine, which effectively attenuate neuroinflammatory cascades (Wang et al., 2021).

It is hypothesized that the therapeutic effect of VNS is mediated, at least in part, via the Cholinergic Anti-inflammatory Pathway (CAP) (Wang et al., 2021). The activation of vagal afferent fibers is capable of modulating the immune response by inhibiting the synthesis of pro-inflammatory mediators.

If VNS effectively mitigates seizures by suppressing neuroinflammation, the dynamics of specific blood protein levels could serve as a reliable tool for biological monitoring. Currently, the evaluation of VNS efficacy relies predominantly on traditional clinical and instrumental parameters (seizure diaries and electroencephalography), which possess limited value for long-term prognosis. Given the cumulative nature of neuromodulation, clinical improvement often exhibits a delayed onset, and the dynamics of EEG patterns do not always directly correlate with the actual therapeutic response. During this period of gradual “effect accumulation,” standard assessment methods may fail to capture the true extent of the ongoing pathophysiological restructuring.

While EEG remains the paramount ancillary diagnostic tool in epilepsy-playing a decisive role in the diagnosis, classification, and localization of epileptogenic foci-its clinical application presents several inherent limitations (Kenzhebekova et al., 2017; Hao and Luo, 2024; Wang et al., 2025).

First, routine scalp EEG possesses relatively low spatial resolution. This modality assesses the electrical activity of only superficial cortical structures (approximately one-third of the cerebral cortex surface area), which significantly reduces its sensitivity in detecting deep-seated epileptogenic generators (Amin and Benbadis, 2019).

Second, interictal epileptiform activity frequently exhibits a sporadic and unpredictable character. Studies indicate that the detection rate of specific discharges during standard routine EEG is only 40%–50%. Even prolonged 24-h EEG monitoring increases the detection frequency to merely 70%–80% (Hao and Luo, 2024). Consequently, the absence or presence of epileptiform activity during a specific timeframe does not allow for a reliable assessment of true disease severity (Xiong, 2023).

Third, the inherent subjectivity in the visual interpretation of data remains a significant challenge. The inter-rater reliability among experts evaluating EEG recordings does not exceed 76% (Jing et al., 2020).

The integration of molecular biomarkers, analogous to modern approaches in oncology or cardiology, would allow clinicians to look beyond visible clinical symptoms and objectify the treatment response.

The search for reliable peripheral biomarkers of neuroinflammation is fraught with methodological challenges. The clinical utility of classical pro-inflammatory mediators (such as IL-1β) is frequently limited by their high kinetic lability in the systemic circulation and their susceptibility to rapid, non-specific fluctuations (Pitkänen et al., 2016). The transient circulating profile of these molecules complicates the assessment of their true diagnostic value and often yields contradictory results. Consequently, current research is focused on identifying proteins that demonstrate greater stability in biological fluids and are suitable for routine, cost-effective monitoring.

The pathophysiology of epilepsy is a multifaceted process, and the current literature describes a wide spectrum of biomarkers reflecting its various components. These include proteins indicative of glial cell activation, such as S100B and glial fibrillary acidic protein (GFAP), which point to reactive astrogliosis.

However, a critical methodological challenge in peripheral biomarker research is tissue specificity. Ideally, inflammatory molecules measured in the blood must be strictly specific to the central nervous system (CNS), ensuring that their analysis is not confounded by non-specific release from peripheral sources. The importance of identifying such highly specific indicators for drug-resistant epilepsy has been highlighted in previous literature (Pollard et al., 2013).

Given that drug resistance is fundamentally driven by ongoing structural neuronal damage and the resulting sterile neuroinflammation, our study was deliberately focused on markers that quantitatively reflect these exact two interrelated processes: neuronal death and the initiation of the subsequent inflammatory cascade. Adhering to the strict criterion of CNS specificity, three proteins with proven pathogenetic significance were selected as our key molecular targets.

Ubiquitin C-terminal hydrolase L1 (UCH-L1) is a highly specific cytoplasmic enzyme, representing one of the most abundantly expressed proteins in neurons (Elhady et al., 2021; Giovannini and Meletti, 2023; Yasak et al., 2020). Its’ physiological significance extends far beyond that of a passive structural indicator. UCH-L1 serves as a key regulator of the ATP-dependent ubiquitin-proteasome system (UPS), ensuring intracellular protein homeostasis (Dong et al., 2023; Chatziefstathiou et al., 2024). Possessing complex enzymatic activity—primarily deubiquitinating (essential for recycling the free ubiquitin pool), alongside ubiquitin ligase functions (Yasak et al., 2020; Bishop et al., 2016)-the enzyme effectively prevents the intracellular accumulation of neurotoxic protein aggregates.

Recurrent epileptic seizures initiate a cascade of pathological changes triggered by excitotoxicity and post-ictal metabolic stress. Local tissue hypoxia induces protein misfolding and the subsequent accumulation of aggregates. Concurrently, driven by concomitant neuroinflammation and oxidative stress, neuronal phospholipid membranes degrade, and the endothelial tight junctions of the blood–brain barrier (BBB) are disrupted (Day and Thompson, 2010). As a result of these structural damages, UCH-L1 passively translocates from the neuronal cytoplasm into the systemic circulation. Due to its strict neuronal specificity and minimal peripheral expression, the detection of UCH-L1 in the blood reliably indicates brain tissue injury and is strongly associated with neuroinflammation, trauma, and compromised BBB permeability (Bishop et al., 2016) (Elhady et al., 2021; Giovannini and Meletti, 2023; Mondello et al., 2012).

The cellular efflux of UCH-L1 and the subsequent depletion of its intracellular pool have critical implications for neuronal viability. The loss of this enzymatic activity leads to the functional failure of the UPS (Yasak et al., 2020; Chatziefstathiou et al., 2024). Under conditions of post-ictal stress, the adequate functioning of this system is crucial for facilitating endogenous neuroprotective mechanisms (Liu et al., 2019). The failure to clear misfolded proteins impairs synaptic transmission renders the neuron highly susceptible to secondary injury, and perpetuates the vicious cycle of irreversible neurodegeneration (Gong et al., 2006).

The clinical utility of UCH-L1 as a translational biomarker has been convincingly demonstrated in several studies. For instance, a comparative analysis of UCH-L1 levels in 160 patients with epilepsy and 100 healthy volunteers revealed a significant, greater than twofold elevation of the enzyme in the epilepsy cohort (8.30 ng/mL vs. 3.90 ng/mL; *p* < 0.001) (Yasak et al., 2020). Crucially for understanding the underlying pathogenesis, biomarker levels were independent of recent seizure activity: no statistically significant difference was observed between patients in the ictal (with recent seizures, 8.50 ng/mL) and interictal (8.10 ng/mL) periods (*p* = 0.612). The consistently elevated marker levels during the interictal period justify its utility for assessing baseline structural deficits and monitoring the restoration of barrier function during therapy, independent of seizure frequency (Banote et al., 2022). This finding corroborates that elevated UCH-L1 does not merely reflect a transient release in response to an acute seizure, but rather a persistent, chronic disruption of BBB permeability.

Furthermore persistently elevated UCH-L1 levels despite optimal medical therapy are significantly associated with active, recurrent seizures. This provides a strong rationale for utilizing this marker to assess baseline structural deficits, monitor disease severity, and facilitate the early identification of patients with drug-resistant epilepsy who require surgical or neuromodulatory interventions, as well as to objectively track the restoration of barrier functions during therapy (Elhady et al., 2021).

The selection of high mobility group box 1 (HMGB1) as a key biomarker is dictated by its fundamental role in the pathogenesis of drug-resistant epilepsy, where it acts as a mechanistic link between excitotoxicity and neuroinflammation. Under conditions of excessive neuronal activity, HMGB1 is either actively secreted or passively released into the extracellular space, functioning as a damage-associated molecular pattern (DAMP) or “alarmin” (Maroso et al., 2010; Paudel et al., 2018). The primary target of extracellular HMGB1 is microglia, the central effector of the brain’s immune system. By binding to Toll-like receptor 4 (TLR4) on the microglial surface, HMGB1 initiates a pro-inflammatory cascade that sustains neuronal hyperexcitability and perpetuates the vicious cycle of epileptogenesis (Shi et al., 2018; Vezzani et al., 2011).

The pathophysiological significance of HMGB1 as a systemic biomarker is further corroborated by its intracellular transport mechanisms. According to Pitkänen et al. (2016), the translocation of this protein from the nucleus to the cytosol is an energy-demanding process that increases neuronal oxygen demand, directly correlating with altered T2 relaxation signals on neuroimaging. Thus, the temporal dynamics of HMGB1 in peripheral blood serve as a robust translational indicator, reflecting not only the magnitude of neuroinflammation but also the degree of metabolic exhaustion within epileptogenic networks.

A defining characteristic of HMGB1’s utility as a biomarker is its correlation with disease severity and pharmacoresistance, rather than merely the occurrence of an acute seizure. Authors have established that patients with drug-resistant epilepsy exhibit significantly elevated levels of protein compared to those with controlled seizures (Nugrahanto et al., 2024).

Serum HMGB1 levels were significantly higher in the patient cohort, with a median value of 6.047 μg/L (4.65–11.05) compared to 2.093 μg/L (1.41–3.25) in the healthy control group. Furthermore, the median blood HMGB1 level was substantially higher in the drug-resistant group [14.26 μg/L, interquartile range (IQR): 9.30–14.26] compared to the drug-responsive group (4.88 μg/L, IQR: 4.65–5.12) (Sakhr et al., 2025; Walker et al., 2022).

A critical aspect of data interpretation concerns protein isoforms. It is well-established that the disulfide isoform of HMGB1 possesses specific agonistic activity toward TLR4. In the present study, in strict accordance with standard clinical practice, the total HMGB1 pool was quantified using an enzyme-linked immunosorbent assay (ELISA) (Sakhr et al., 2025).

Because isoform differentiation is a costly procedure not integrated into routine laboratory monitoring, the utilization of validated assays for total HMGB1 remains an international standard that ensures high reproducibility. Given that the pathogenic fraction is the primary driver of inflammation, a statistically significant reduction in total HMGB1 levels inherently reflects the suppression of the pro-epileptogenic process. (Yue et al., 2023; Ulfa et al., 2024).

The translational value of the HMGB1/TLR4 axis is most profoundly realized in the context of neuromodulation, as microglia play a central role in the cholinergic anti-inflammatory mechanisms of VNS (Namgung et al., 2022; Chen et al., 2021). It has been demonstrated that the VNS-induced elevation of acetylcholine within brain structures leads to the targeted activation of α7-nicotinic acetylcholine receptors (α7nAChR) on microglial cells. The activation of α7nAChR triggers an intracellular cascade—including the stimulation of adenylate cyclase 6—that induces the phenotypic shift of microglia from an aggressive (amoeboid) to a surveillance state. This transition is accompanied by the inhibition of the NF-κB pathway and a subsequent decrease in cytokine release (IL-1β, IL-6) (Mizrachi et al., 2021). Furthermore, this cascade promotes the physical degradation of the TLR4 receptor complex. (Kim et al., 2014; Zhu et al., 2021). In the context of epileptogenesis, this mechanism is of paramount importance. Because the deleterious effects of HMGB1 are mediated primarily through its interaction with TLR4, the VNS-induced degradation of this receptor essentially deprives the “alarmin” of its primary molecular target. Thus, cholinergic modulation does not merely reduce circulating levels of the inflammatory ligand; it actively interrupts pathological DAMP signaling at the receptor level, thereby conferring a robust neuroprotective effect.

Neuron-specific enolase (NSE) is a classic, highly specific marker of structural damage to the central and peripheral nervous systems, serving as a reliable prognostic indicator in various neurological disorders, including epilepsy (Tikhonova et al., 2024; Maiti et al., 2017; Rakhimbaeva and Rashidova, 2011).

Plasma NSE levels strictly correlate with disease severity and seizure frequency, objectively reflecting the extent of ongoing neurodegeneration. The clinical utility of this protein is corroborated by multiple studies; notably, a direct correlation has been demonstrated between serum NSE concentrations and clinical severity, with peak marker levels observed in patients experiencing the highest frequency of paroxysms (Correale et al., 1998). The relevance of monitoring NSE specifically in pediatric practice is further substantiated by a recent meta-analysis which synthesized data from pediatric epilepsy studies and verified the diagnostic value of elevated serum NSE titers as a robust indicator of neuronal distress (Mu et al., 2020).

Ideally, as noted by Pitkänen, molecules measured in the systemic circulation must be strictly CNS-specific to ensure their analysis is not confounded by non-specific release from peripheral sources. Furthermore, according to the criteria outlined in the WONOEP expert review, an ideal peripheral biomarker should possess not only high brain tissue specificity but also the capacity for extravasation upon blood–brain barrier (BBB) disruption (Walker et al., 2016).

The significance of NSE as a monitoring tool is additionally reinforced by its sensitivity to therapeutic interventions. According to controlled trials the temporal dynamics of serum NSE during conservative anti-seizure therapy are directly contingent upon the patient’s clinical response. Specifically, it has been shown that NSE levels remain persistently elevated in cohorts where conservative treatment fails, reflecting ongoing tissue destruction (Maiti et al., 2017; Chen et al., 2021; Mondello et al., 2012).

The working hypothesis postulates that successful VNS therapy modulates neuroinflammation and attenuates excitotoxicity, effects that are quantitatively reflected by a decline in HMGB1, NSE, and UCHL-1 levels, correlating with a clinical reduction in seizure frequency.

### 1.1 Study objective

To evaluate the prognostic value and longitudinal dynamics of inflammatory and neurodegenerative biomarkers (HMGB1, NSE, UCHL-1) in pediatric patients with drug-resistant epilepsy undergoing VNS therapy.

## 2 Materials and methods

### 2.1 Study design

This study presents a longitudinal clinical analysis of 40 pediatric patients with drug-resistant structural focal epilepsy. The overall cohort was categorized into an active VNS intervention arm (*n* = 20) and a parallel comparison arm (*n* = 20) receiving ongoing optimal medical therapy (OMT). To ensure that the observed biomarker dynamics were associated with VNS therapy rather than natural disease progression, parallel longitudinal assessments were conducted in the medically treated cohort. As the primary objective of this article is to elucidate the neuroimmune mechanisms underlying neuromodulation, the detailed intra-individual trajectory modeling, correlation analyses, and responder stratification are focused specifically on the VNS intervention group.

The detailed clinical and demographic characteristics of the active VNS cohort (*n* = 20) analyzed in this study are presented in Table 1.

**Table 1:** Demographic and clinical characteristics of the study cohort (*n* = 20).

Characteristics	Value
Sex, *n* (%)	Male: 12 (60%)/Female: 8 (40%)
Age at surgery, years	6.5 [4; 9]
Median [Min; Max]	
Diagnosis (ILAE 2017). Structural focal epilepsy	20 (100%)
Predominant seizure type:	20 (100%)
MRI characteristics/structural etiology, *n* (%):20 (100%)	20 (100%)
Sequelae of perinatal CNS injury	9 (45%)
Post-hypoxic encephalopathy, periventricular leukomalacia (PVL), gliotic-atrophic and cicatricial changes	
Malformations of cortical development (MCD)	7 (35%)
Malformations of cortical development: Focal Cortical Dysplasia (FCD), pachygyria, megalencephaly, Corpus callosum dysgenesis, Hippocampal inversion	
Combined and other structural anomalies	4 (20%)
Arachnoid & porencephalic cysts, hippocampal sclerosis. Ventriculomegaly/Hydrocephalus	
Surgical status: non-resectable	20 (100%)
Reason for ineligibility: inability to perform radical resection due to multifocality, diffuse lesion extent with ill-defined boundaries, or involvement of extensive eloquent cortical areas.	
Duration of epilepsy, years	4.5 [2; 8]
Median [Min; Max]	
Number of ASMs	2 [2; 4]
Median [Min; Max]	
Baseline seizure frequency (per month)	13.5 [9; 48]
Median [Min; Max]	

The predominant seizure phenotype was focal to bilateral tonic–clonic seizures. The secondary bilateralization of seizure activity contributed to the clinical severity and the systemic nature of the neuroinflammatory response.

Despite the presence of MRI-confirmed structural abnormalities (structural etiology), patients were deemed ineligible for resective surgery. Contraindications to resection included: diffuse lesion extent (e.g., post-hypoxic–ischemic changes, gliosis), ill-defined epileptogenic zone boundaries, multifocality, or the involvement of eloquent cortical areas.

Consequently, VNS therapy was selected as the optimal modality for palliative neuromodulation in patients with verified structural brain pathology but contraindications to open surgery.

All patients received optimal medical therapy (OMT) comprising a combination of appropriate broad-spectrum anti-seizure medications (predominantly valproic acid, levetiracetam, topiramate, or lamotrigine). These specific ASM regimens were carefully tailored to each patient’s specific epilepsy syndrome, seizure semiology, and individual tolerability prior to study enrollment. During the 12-month observation period, the core pharmacological combinations were maintained without the introduction of novel drugs or the complete withdrawal of baseline medications. Routine dose adjustments were performed in accordance with standard pediatric clinical practice, primarily to account for physiological weight gain or to address transient fluctuations in seizure frequency. This approach allowed for necessary clinical flexibility while preserving a consistent pharmacological background across the entire study duration.

#### 2.1.1 Inclusion criteria

*Age*: 4 to 9 years (inclusive) at the time of enrollment.

*Epilepsy type*: Confirmed diagnosis of drug-resistant focal epilepsy (in accordance with the ILAE 2017 classification).

*Predominant seizure type*: Presence of persistent focal to bilateral tonic–clonic seizures as the dominant seizure phenotype.

*Pharmacoresistance*: Documented failure of two or more appropriately chosen and administered anti-seizure medication (ASM) schedules (whether as monotherapies or in combination), meeting the ILAE consensus definition of drug-resistant epilepsy.

*Stable therapeutic regimen*: Maintenance of stable ASM doses for a minimum of 3 months prior to device implantation.

*Surgical ineligibility*: Absence of a clearly circumscribed epileptogenic focus amenable to resection (e.g., MRI-negative cases) or the presence of multifocal lesions.

*Informed consent*: Written informed consent obtained from the patient’s legal guardians/representatives.

#### 2.1.2 Exclusion criteria

*Progressive CNS disorders*: Diagnosed neurodegenerative or metabolic disorders, or the presence of intracranial space-occupying lesions.

*Psychogenic seizures*: Documented history of psychogenic non-epileptic seizures (PNES).

*Biomarker confounding factors*: Presence of acute infectious pathology, autoimmune diseases, or chronic inflammatory processes in the acute phase (including neuroinfections and systemic connective tissue disorders) capable of confounding systemic HMGB1, NSE, or UCHL-1 levels.

*Severe somatic comorbidities*: Decompensated cardiovascular diseases (specifically cardiac arrhythmias or conduction abnormalities), or severe hepatic or renal insufficiency.

*Surgical history*: Prior resective epilepsy surgery or the implantation of other neurostimulation systems.

*Anatomical contraindications*: Prior vagotomy or anatomical anomalies precluding electrode implantation on the left vagus nerve.

*Compliance*: Anticipated inability of the patient or their legal guardians to adhere to the study protocol and follow-up visit schedule.

### 2.2 Study methods

Patient evaluation entailed a standard neurological examination and a detailed analysis of epilepsy characteristics.

Therapeutic efficacy was assessed based on seizure diaries maintained by the patients’ legal guardians. Key efficacy endpoints included changes in mean monthly seizure frequency and seizure severity dynamics. To objectively quantify the latter, the National Hospital Seizure Severity Scale (NHS-3) was utilized.

The diagnostic workup comprised prolonged video-EEG monitoring (to verify seizure type and assess interictal activity) and high-field brain MRI acquired using a dedicated epilepsy protocol.

#### 2.2.1 Stimulation parameter programming and titration protocol

Selection of Stimulation Parameters (Months 0–3) Standard settings were selected as the baseline parameters for therapy initiation: a signal frequency of 30 Hz and a pulse width of 500 μs. This selection was predicated on the necessity of achieving maximal recruitment of vagal afferent fibers in patients with drug-resistant epilepsy.

According to dosing guidelines, the combination of a 500 μs pulse width and a 30 Hz frequency enables the attainment of a clinically significant therapeutic threshold at an output current as low as 1.5 mA. However, to maximize the anti-seizure effect, our protocol stipulated titration up to the upper limit of the recommended therapeutic range (1.75–2.25 mA).

Particular attention was paid to the monitoring of tolerability. In the event of clinically significant dose-dependent adverse events (hoarseness, dysphonia, coughing, or cervical discomfort), a temporary suspension of titration strategy was employed. In such instances, further current escalation was temporarily suspended for a period of 7 days to facilitate patient habituation to the current parameters. Following adaptation, titration was resumed until target values were achieved according to the standard protocol (Orosz et al., 2014; LivaNova USA, Inc., 2023).

In the event of persistent adverse effects, the titration step was deferred for an additional week. Consequently, the interval between parameter escalations varied from 1 to 2 weeks, thereby ensuring the attainment of the target dose without compromising patient compliance.

The study design, featuring assessment time points at 3, 6, and 12 months, was selected to evaluate the distinct mechanisms of action of VNS therapy:

*Month 3*: This time point marked the completion of the active current titration phase. In accordance with manufacturer recommendations, rapid titration to the target output current within the initial 3-month period facilitates a faster onset of clinical response. At this juncture, the minimally effective output current was established, and the therapeutic strategy shifted from amplitude escalation to increasing the duty cycle.

*Month 6*: By this interval, patients had undergone the initial duty cycle increments (reaching 15 and 23%). This time point permitted the evaluation of therapy intensification efficacy in patients who achieved only a partial response to standard stimulation. Furthermore, guidelines recommend that duty cycle adjustments be implemented no sooner than 3–6 months’ post-stabilization, a recommendation with which our protocol remains in alignment. A study involving a large pediatric cohort (*n* = 347) demonstrated that the first statistically significant response level is established by month 6 (approximately 32.5% responders) (Orosz et al., 2014).

*Month 12*: The final observation point corresponded to the transition to a 30% duty cycle regimen. This allowed for the assessment of the long-term cumulative effect of neuromodulation and the tolerability of aggressive stimulation parameters (7 s ON/30 s OFF) over an extended period. Data from the same study indicate that the responder rate continues to rise from 32.5% (at 6 months) to 37.6% (at 12 months) and beyond (Orosz et al., 2014).

### 2.3 Laboratory methods

Quantitative determination of plasma biomarker concentrations was performed using enzyme-linked immunosorbent assay (ELISA).

Commercial assay kits were utilized for the detection of:

Ubiquitin C-terminal Hydrolase L1 (UCH-L1);

High Mobility Group Box 1 (HMGB1);

Neuron-Specific Enolase (NSE).

All laboratory procedures were conducted in strict adherence to the manufacturer’s protocols (Thermo Fisher Scientific, United States).

In accordance with the manufacturer’s instructions, blood samples were centrifuged at 1000×g for 15 min at 2–8 °C within 30 min of collection. The resulting plasma was aliquoted and stored frozen at −80 °C until subsequent analysis. Samples were run in duplicate. The intra-assay and inter-assay coefficients of variation were <10% and <15%, respectively.

*Human High Mobility Group Protein B1 (HMGB1) Sample Preparation and Pre-Analytical Phase*: Venous blood samples were collected into tubes containing EDTA as an anticoagulant. ELISA Protocol: The analysis was performed using a sandwich ELISA method in strict adherence to the protocol.

*Neuron-Specific Enolase (NSE) Test System Specification*: The Human Neuron Specific Enolase ELISA Kit was utilized for the quantitative determination of NSE levels. Assay Protocol: The analysis was conducted in accordance with the manufacturer’s instructions. Repeated freeze–thaw cycles and the use of hemolyzed plasma were strictly excluded. Matrix Justification: EDTA plasma was selected as the biological matrix for analysis. This sample type minimizes the risk of false-positive results associated with *in vitro* hemolysis and the release of NSE from blood cellular elements, which aligns with pre-analytical recommendations for this specific biomarker.

*Ubiquitin C-terminal Hydrolase L1 (UCH-L1) Test System Specification*: The Human UCH-L1 ELISA Kit was employed for the quantitative assessment of UCH-L1 levels. Sample Dilution: Prior to analysis, in accordance with the instructions, thawed plasma samples were diluted at a 1:2 ratios (2-fold dilution) using Assay Diluent C.

### 2.4 Statistical data analysis

Statistical data analysis was performed using the Statistica 8.0 software package (StatSoft Inc., United States).

The normality of the distribution of quantitative variables was assessed using the Shapiro–Wilk test. Given the small sample size (*n* = 20) and the non-normal distribution of the data, descriptive statistics are presented as the median and interquartile range (Me [Q1; Q3]).

To analyze the longitudinal dynamics of neurospecific biomarker concentrations (UCH-L1, HMGB1, NSE) across four related time points (baseline, 3, 6, and 12 months), the non-parametric Friedman test was employed.

Upon detection of statistically significant differences (*p* < 0.05), post-hoc pairwise comparisons were conducted using Dunn’s test with adjustment for multiple comparisons. Differences were considered statistically significant at *p* < 0.05.

## 3 Results

### 3.1 Longitudinal dynamics and statistical analysis

Table 2 illustrates the longitudinal dynamics of UCH-L1 concentrations at baseline and at the 3, 6, and 12-month follow-up intervals.

**Table 2:** Descriptive characteristics of plasma UCH-L1 levels.

Time point	0 mo	3 mo	6 mo	12 mo
Median	5.29	4.61	3.73	2.66
Minimum	4.5	2.06	1.95	1.09
Maximum	7.1	7.33	7.03	5.8
Quartile 1	5.09	3.53	3.26	2.18
Quartile 3	5.72	5.48	4.91	3.95

The overall dynamics of UCH-L1 levels demonstrated a progressive decline over the 12-month follow-up period during VNS therapy (Friedman test, *χ*^2^ = 41.34, d*f* = 3, *p* < 0.001). At baseline (Month 0), the median concentration was 5.29 ng/mL (IQR [5.09; 5.72]). Median levels subsequently decreased to 4.61 [3.53; 5.48] ng/mL at 3 months, 3.73 [3.26; 4.91] ng/mL at 6 months, and reached a minimum of 2.66 [2.18; 3.95] ng/mL at the 12-month final time point.

Post-hoc pairwise comparisons (Dunn-Bonferroni test) identified the specific stages of this significant reduction. A significant decrease relative to baseline was first observed by Month 6 (Adj. *p* = 0.009). The most pronounced effect occurred at Month 12, which significantly differed from both the baseline (Adj. *p* < 0.001) and the Month 6 value (Adj. *p* = 0.019). Additionally, the difference between Month 3 and Month 12 was highly significant (Adj. *p* < 0.001), whereas the change between Month 3 and Month 6 did not reach the threshold of statistical significance following Bonferroni correction (Adj. *p* = 0.11).

In contrast, analysis of UCH-L1 levels in the control group (*n* = 20) revealed no statistically significant changes over the 12-month follow-up period (Friedman test, *χ*^2^ = 2.4; d*f* = 3; *p* = 0.494). The median biomarker concentration remained stable across all study time points: 7.79 [6.94; 8.61] ng/mL at baseline, 7.89 [7.02; 8.93] ng/mL at 3 months, 7.85 [7.22; 8.66] ng/mL at 6 months, and 8.07 [7.31; 8.73] ng/mL by the 12-month follow-up.

For an in-depth assessment of the relationship between biomarkers and clinical outcomes, the interventional cohort was stratified into clinical responders (seizure frequency reduction ≥50%, *n* = 8) and non-responders (<50%, *n* = 12). The selection of this 50% stratification threshold is predicated on the consensus criteria of the International League Against Epilepsy (ILAE) for defining a clinically meaningful response. Stratification of clinical outcomes according to the McHugh classification at 12 months revealed the following distribution: Class I was achieved in 4 patients (20%), and Class II in 4 patients (20%). A partial clinical response corresponding to Class III was recorded in 11 patients (55%), while 1 patient (5%) exhibited a Class IV response.

Comparative intergroup analysis was conducted using the nonparametric Mann–Whitney *U* test—where *U* represents the primary test statistic derived from the rank sums of the independent samples, and *Z* denotes the standardized normal deviate (*Z*-score) utilized to approximate the exact significance level. This methodology enabled the evaluation of the diagnostic utility of absolute marker concentrations at fixed temporal endpoints.

The analysis revealed no statistically significant differences in baseline UCH-L1 concentrations (Month 0) between the two subcohorts (*U* = 38.0; *Z* = 0.77; *p* = 0.440), thereby confirming the baseline comparability of the groups prior to therapy initiation. However, by the 12-month follow-up, UCH-L1 levels were highly significantly lower in the clinical responder subcohort compared to the non-responders (*U* = 0.00; *Z* = 3.70; *p* = 0.0002).

**Figure 1:**
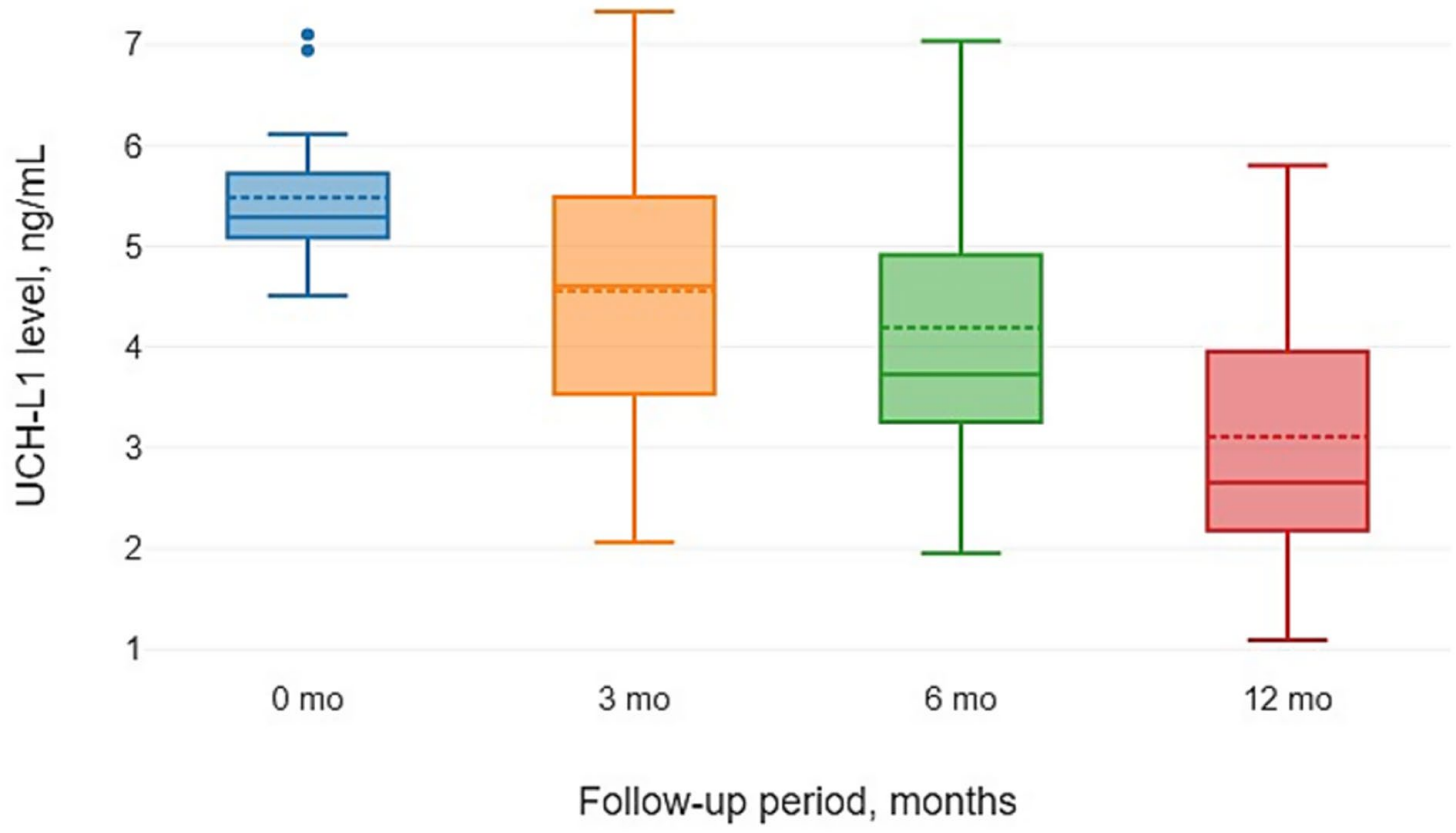
Progressive reduction in plasma UCH-L1 concentrations during VNS therapy. The box plots demonstrate a consistent decline in median biomarker levels from 5.29 ng/mL at baseline (0 mo) to 4.61 ng/mL (3 mo), 3.73 ng/mL (6 mo), and 2.66 ng/mL at the 12-month follow-up. Statistical significance was confirmed via the Friedman test (*p* < 0.001) with post-hoc Dunn-Bonferroni corrections indicating significant reductions by Month 6 and Month 12.

Figure 1 illustrates a progressive narrowing of the interquartile range and a reduction in median levels, reflecting the stabilization of neurochemical biomarkers under VNS therapy.

The analysis of clinical seizure frequency dynamics during VNS therapy revealed statistically significant differences (Friedman test, *χ*^2^ = 42.85, *p* < 0.001). The baseline median seizure frequency was 13.5 [10.75; 16.25] per month. By Month 6 of therapy, the median had declined to 9.0 [7.0; 16.0], and by Month 12, it reached 7.5 [4.75; 11.0] which represents a 44.4% reduction from the baseline. In contrast to the VNS intervention group, the comparison group exhibited no longitudinal dynamics in seizure frequency over the 12-month follow-up period (Friedman test, *χ*^2^ = 0.46, *p* = 0.927). The median seizure frequency remained stable throughout the entire study period, varying between 13.0 and 13.5 per month.

The detailed descriptive statistics summarizing these longitudinal changes are presented in Table 3.

**Table 3:** Changes in median monthly seizure frequency over the 12-month follow-up period.

Time point	0 mo	3 mo	6 mo	12 mo
Median	13.5	13.5	9	7.5
Minimum	9	6	4	1
Maximum	48	38	37	31
Quartile 1	10.75	8	7	4.75
Quartile 3	16.25	17	16	11

Data are presented as median, range (Min–Max), and quartiles (Q1; Q3).

To identify the specific time intervals associated with the most significant reduction, Dunn’s test with Bonferroni correction was conducted. The analysis yielded the following results: First 3 months: No statistically significant differences compared to baseline were observed (Adj. *p* = 1.000). At 6 months: A statistically significant reduction in seizure frequency relative to baseline was recorded (0 mo vs. 6 mo: Adj. *p* = 0.023). While a downward trend was observed compared to Month 3, it did not reach the threshold of statistical significance (Adj. *p* = 0.200). At 12 months: The most pronounced clinical effect was observed. The reduction in seizure frequency at Month 12 was highly significant compared to baseline (Adj. *p* < 0.001), as well as compared to the results at Month 3 (Adj. *p* < 0.001) and Month 6 (Adj. *p* = 0.005).

**Figure 2:**
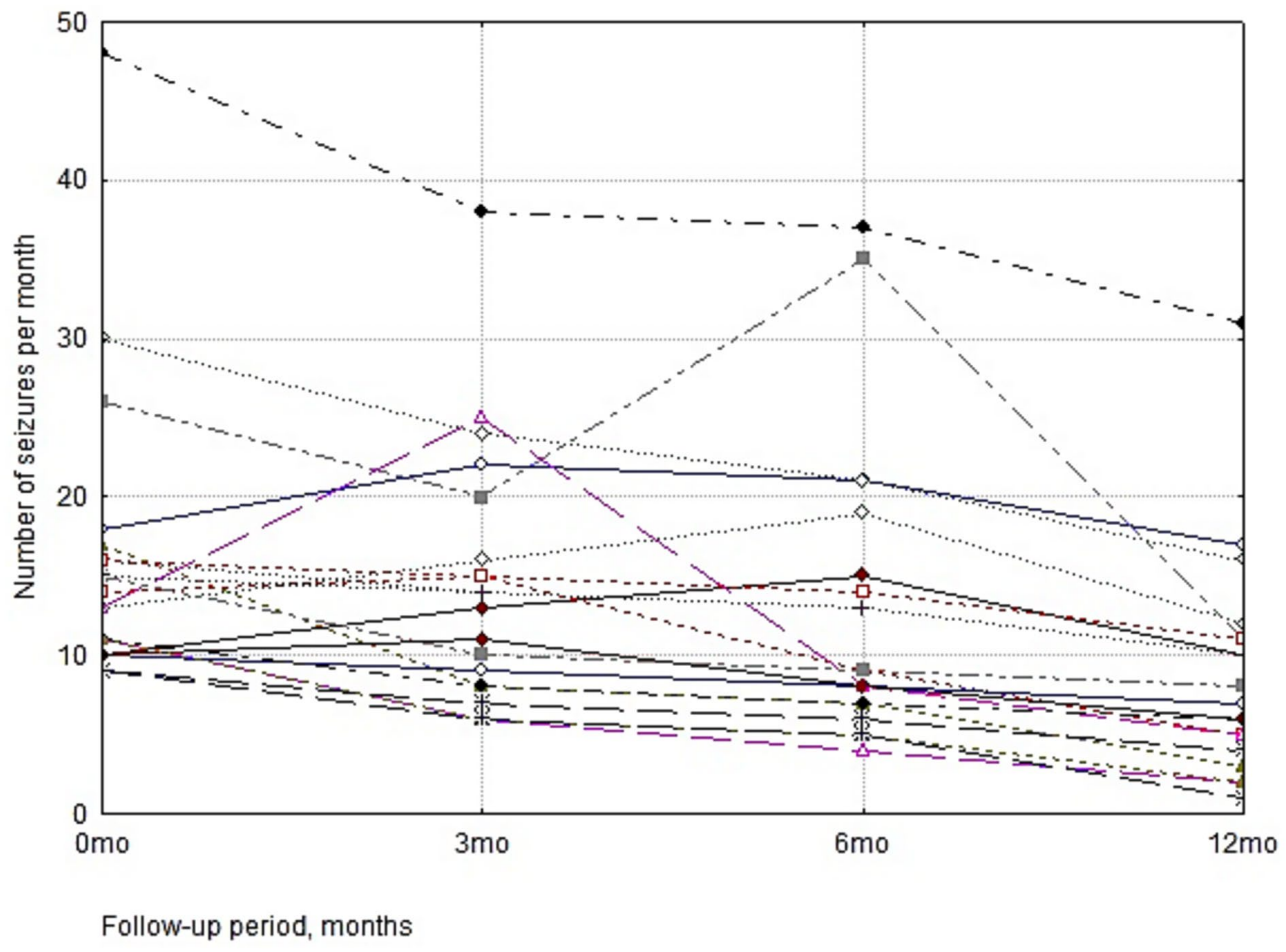
Individual trajectories of monthly seizure frequency over a 12-month VNS therapy follow-up. The line chart displays intra-individual dynamics for each patient in the active intervention arm (*n* = 20). The plot demonstrates both a progressive reduction in absolute seizure counts and a marked narrowing of inter-individual variability by the end of the observation period.

Furthermore, as illustrated in Figure 2, a substantial narrowing of the seizure frequency range was recorded.

**Figure 3:**
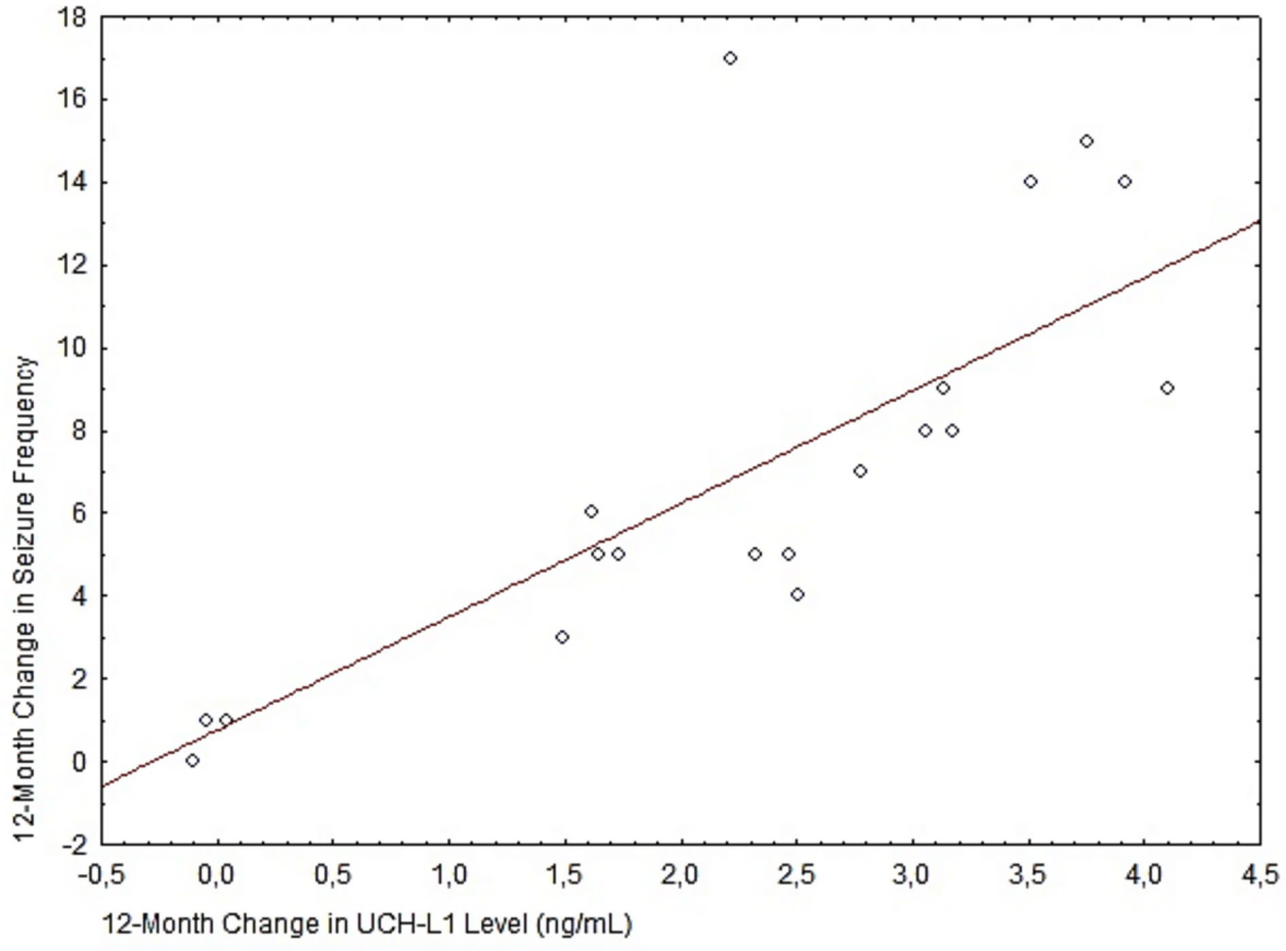
Scatterplot illustrating the correlation between biochemical and clinical response. The graph shows the relationship between the 12-month change in inflammatory marker levels (*x*-axis) and the concomitant change in seizure frequency (*y*-axis). The red line indicates the linear trend (*r* = 0.79 *p* < 0.05).

A key finding was the establishment of a robust interrelationship between biochemical and clinical parameters. Spearman’s correlation analysis, as illustrated in Figure 3, revealed a strong positive association between the reduction in neurospecific marker levels and the decrease in seizure frequency (Spearman’s correlation, *r* = 0.79; *p* < 0.05). The obtained correlation coefficient indicates that favorable biochemical changes in the blood occur synchronously with clinical improvement.

In addition to the clinical reduction in seizure frequency, VNS therapy resulted in significant improvements in electroencephalographic parameters (Table 4).

**Table 4:** Analysis of longitudinal electrophysiological data revealed a pronounced restructuring of epileptic activity following 12 months of VNS therapy.

EEG parameter	Baseline (month 0)	Follow-up (month 12)
High/very high index of epileptiform activity	10 (50%)	5 (25%)
Moderate index of epileptiform activity	10 (50%)	4 (20%)
Low/low-to-moderate index of epileptiform activity	0 (0%)	11 (55%)
Clinical seizures recorded during the EEG epoch	4 (20%)	1 (5%)
Presence of subclinical electrographic patterns	16 (80%)	19 (95%)

At baseline, 100% of patients in the interventional cohort (*n* = 20) exhibited a high or moderate index of interictal epileptiform activity. After 12 months of neuromodulation, a significant de-escalation of the electrical burden was observed: more than half of the patients (55%, *n* = 11) demonstrated a reduction in the epileptiform activity index to “low” or “low-to-moderate” levels.

**Figure 4:**
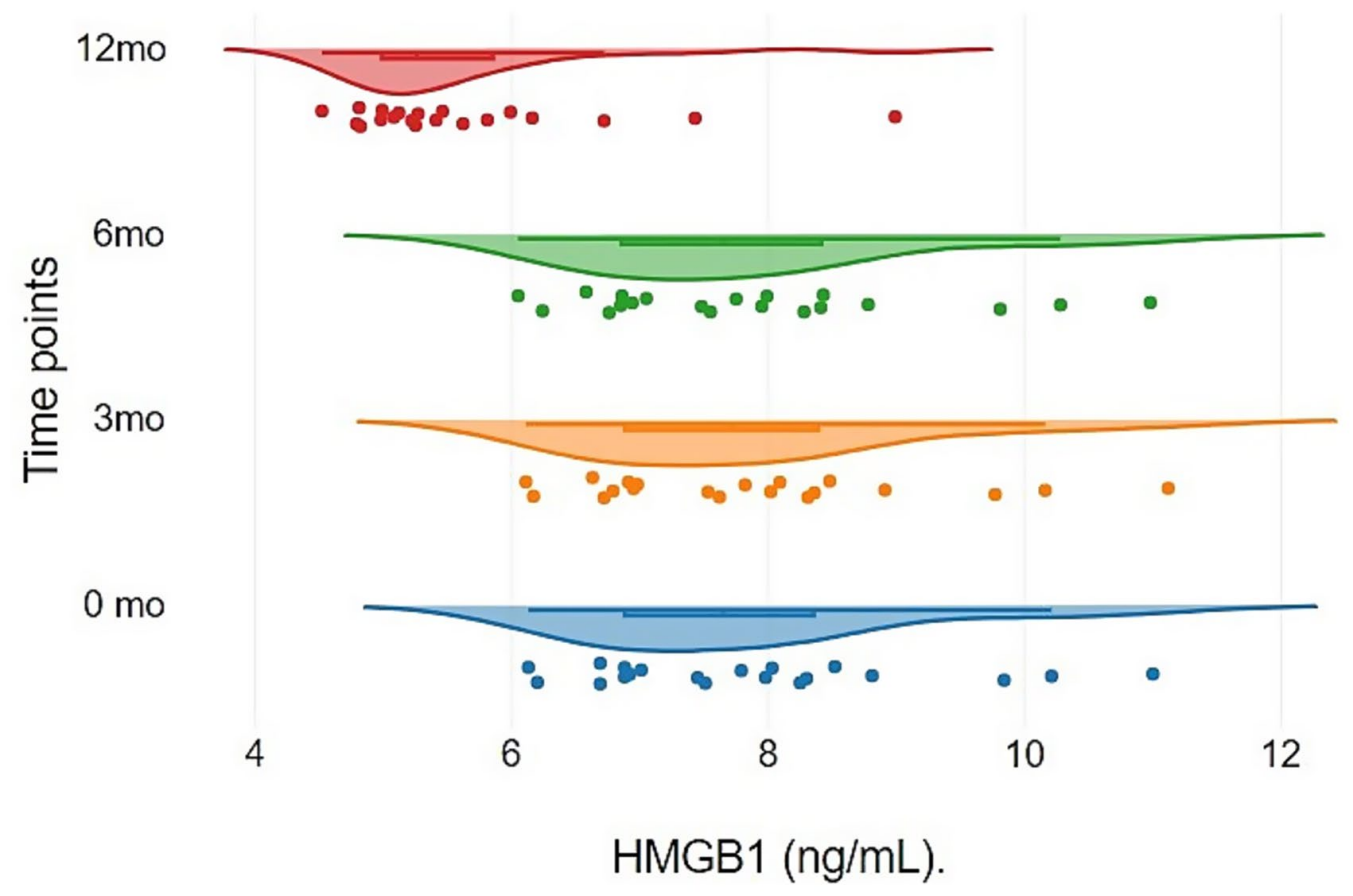
Temporal profile of plasma HMGB1 concentrations during VNS therapy. Raincloud plots illustrate the longitudinal distribution of biomarker levels (ng/mL) at baseline, 3, 6, and 12 months. The visualization highlights a prolonged phase of stability during the initial 6 months, followed by a significant reduction in both absolute concentration and inter-individual variability at the 12-month follow-up.

The dynamics of plasma HMGB1 levels were characterized by a prolonged period of stability: no significant deviations from baseline values were recorded during the first 6 months of therapy (*p* > 0.05). However, as illustrated in Figure 4. By month 12 of follow-up, a marked statistically significant reduction in marker concentration was observed in 90% of the patients. The established moderate correlation between this reduction and the decrease in seizure frequency (*r* = 0.63) allows HMGB1 to be considered a delayed biomarker of VNS therapy efficacy, reflecting long-term neuromodulation. Table 5 shows plasma HMGB1 levels in patients (*n* = 20) at different stages of VNS therapy follow-up.

**Table 5:** Plasma HMGB1 levels in patients (*n* = 20) over the 12-month follow-up period.

HMGB1	0 mo	3 mo	6 mo	12 mo
Median	7.65	7.72	7.65	5.26
Minimum	6.13	6.11	6.05	4.52
Maximum	11	11.12	10.98	8.99
Quartile 1	6.88	6.88	6.86	4.99
Quartile 3	8.36	8.39	8.41	5.86

Data are presented as median, range (Min–Max), and quartiles (Q1; Q3).

The analysis of plasma HMGB1 dynamics revealed stability in marker levels during the first 6 months of therapy. Median concentrations at 0, 3, and 6 months showed negligible differences, varying within a narrow range of 7.65–7.72 ng/mL. However, by Month 12, a marked reduction in HMGB1 levels was observed, reaching a median of 5.26 [4.99; 5.86] ng/mL. The narrowing of the interquartile range at the final time point (from 6.88–8.36 at baseline to 4.99–5.86 at Month 12) confirms the high consistency of the data and the reliability of the marker reduction during the late stages of therapy.

Analysis of biochemical parameters established a statistically significant change in plasma HMGB1 concentrations during VNS therapy (Friedman test, Chi-square = 27.19; d*f* = 3; *p* < 0.001).

To evaluate biomarker dynamics at each therapeutic stage in detail, a post-hoc analysis using the Bonferroni correction for multiple comparisons was performed.

The detailed results of this analysis are presented in Table 6.

**Table 6:** Statistical outcomes of these pairwise comparisons.

Time intervals	Test statistics	Standard error	Std. test statistics	*p*	Adj. *p*
0 mo–3 mo	−0.32	0.41	−0.8	0.426	1
0 mo–6 mo	0.1	0.41	0.24	0.806	1
0 mo–12 mo	1.63	0.41	3.98	<0.001	<0.001
3 mo–6 mo	0.42	0.41	1.04	0.298	1
3 mo–12 mo	1.95	0.41	4.78	<0.001	<0.001
6 mo–12 mo	1.53	0.41	3.74	<0.001	0.001

**Figure 5:**
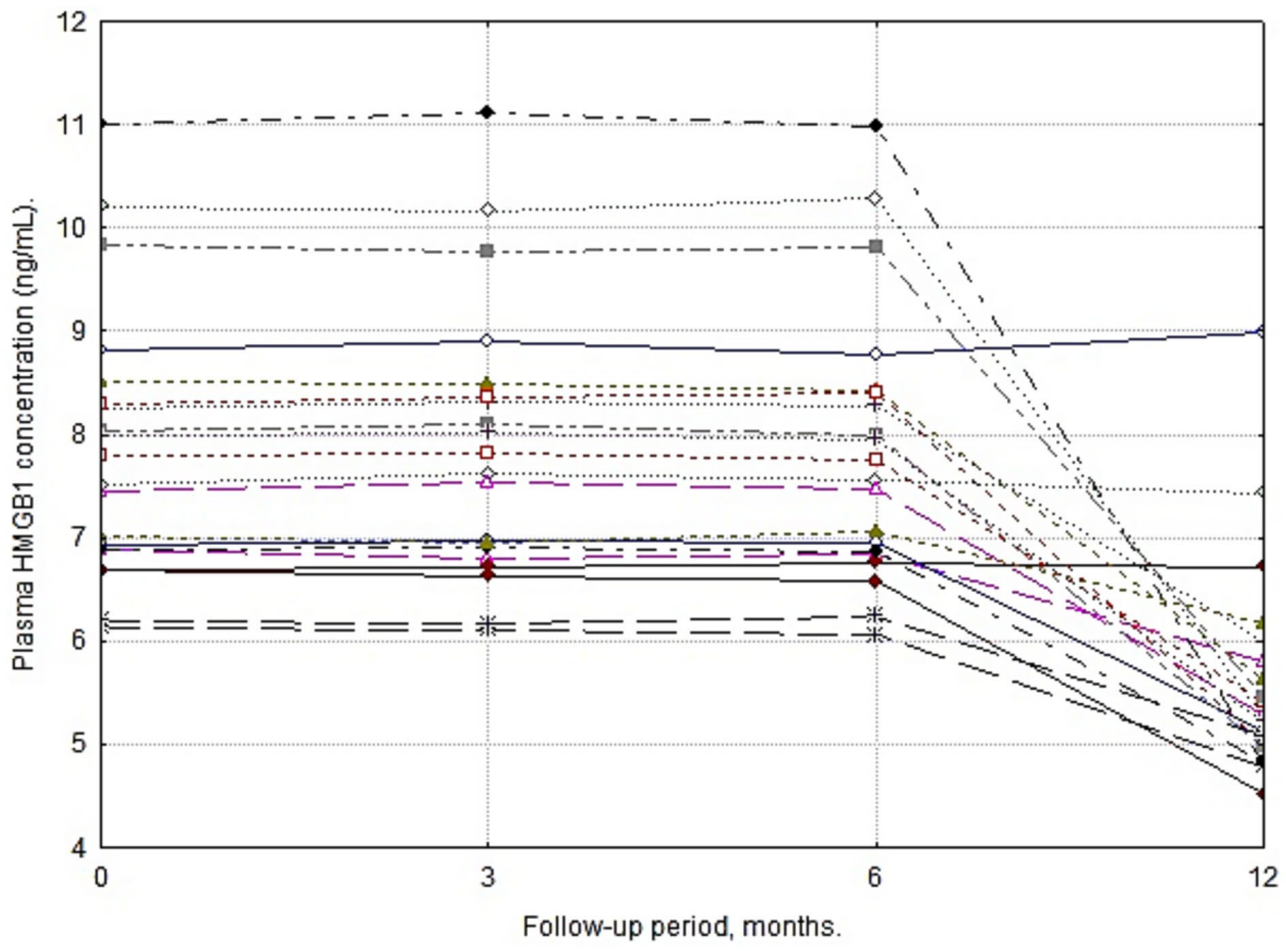
Individual longitudinal trajectories of plasma HMGB1 concentrations during VNS therapy. The line graph illustrates the intra-individual temporal profile of HMGB1 levels (ng/mL) for each patient in the active VNS cohort across the 12-month follow-up. The data demonstrate a prolonged period of biomarker stability during the first 6 months of therapy, followed by a consistent and pronounced reduction in concentration by Month 12. Each line represents a single patient’s continuous measurement.

In pairwise comparisons using the Bonferroni correction, it was established that the HMGB1 concentration at Month 12 was significantly lower than the values at all previous follow-up stages (Adj. *p* ≤ 0.001), whereas differences between Months 0, 3, and 6 were absent, as demonstrated in Figure 5 (Adj. *p* = 1).

The statistical significance of the differences was confirmed for the final follow-up point compared to the baseline level and intermediate stages (Adj. *p* ≤ 0.001 with Bonferroni correction).

In the VNS intervention group, evaluation of HMGB1 levels revealed marked inter-individual variability in baseline values (median 7.65 ng/mL, IQR [6.88; 8.36], range: 6.13–11.0 ng/mL). Because of this profound dispersion, the gradual therapeutic reduction was partially masked when comparing absolute concentrations at fixed time points (Mann–Whitney *U* test, *p* = 0.247). Specifically, patients presenting with initially extreme values demonstrated a robust decline in HMGB1; however, their final absolute concentrations occasionally remained higher than the baseline values of patients with an initially low inflammatory pool. To objectively verify the progressive downward trend while mitigating the confounding influence of initial variability, a Spearman rank correlation analysis of continuous variables was employed, revealing a significant positive association between the magnitude of biomarker reduction and clinical seizure decrease (*r* = 0.63, *p* < 0.05).

**Figure 6:**
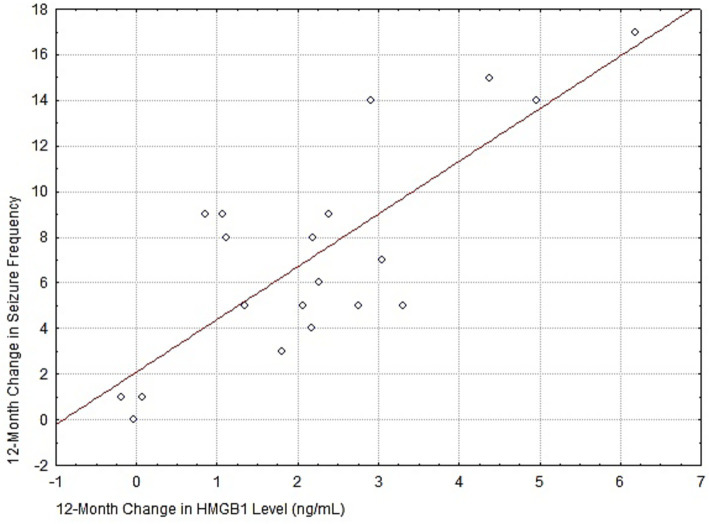
The regression line confirms the general trend: the greater the decrease in the patient’s plasma HMGB1 level, the more pronounced the clinical response in terms of seizure frequency reduction. The presence of several data points near zero values on both axes indicates a subgroup of patients with minimal biochemical and clinical response to the therapy.

The constructed scatterplot Figure 6 demonstrates a moderately strong positive correlation. Spearman’s rank correlation coefficient was *r* = 0.63 (*p* < 0.05).

In Figure 6, the regression line confirms the general trend: the greater the decrease in the patient’s plasma HMGB1 level, the more pronounced the clinical response in terms of seizure frequency reduction. The presence of several data points near zero values on both axes indicates a subgroup of patients with minimal biochemical and clinical response to the therapy.

Analysis of HMGB1 levels in the control group (*n* = 20) revealed no statistically significant changes over the 12-month follow-up period (Friedman test, *χ*^2^ = 3.05; d*f* = 3; *p* = 0.385). The median biomarker concentration remained stable across all study time points: 9.75 [9.27; 10.80] ng/mL at baseline, 10.05 [9.27; 10.94] ng/mL at 3 months, 9.78 [9.32; 10.77] ng/mL at 6 months, and 10.15 [9.55; 10.93] ng/mL by the 12-month follow-up.

NSE levels were characterized by marked heterogeneity. By Month 12 of follow-up, the median level was 1.56 [1.11; 4.78] ng/mL (Table 7). A significant data dispersion persisted, with isolated peak values (up to a maximum of 25.24 ng/mL) recorded in individual patients. The presence of such extreme outliers alongside a consistently low median confirms the absence of a systemic effect of neurostimulation on this parameter.

**Table 7:** Data are presented as median values, minimum-maximum range, and interquartile range (Q1–Q3).

Statistic	0 mo	3 mo	6 mo	12 mo
Median	1.34	0.88	1.37	1.56
Minimum	0.19	0.08	0.18	0.15
Maximum	25.24	21.23	22.16	25.19
Quartile 1	0.88	0.43	0.85	1.11
Quartile 3	5.2	1.38	5.03	4.78

**Figure 7:**
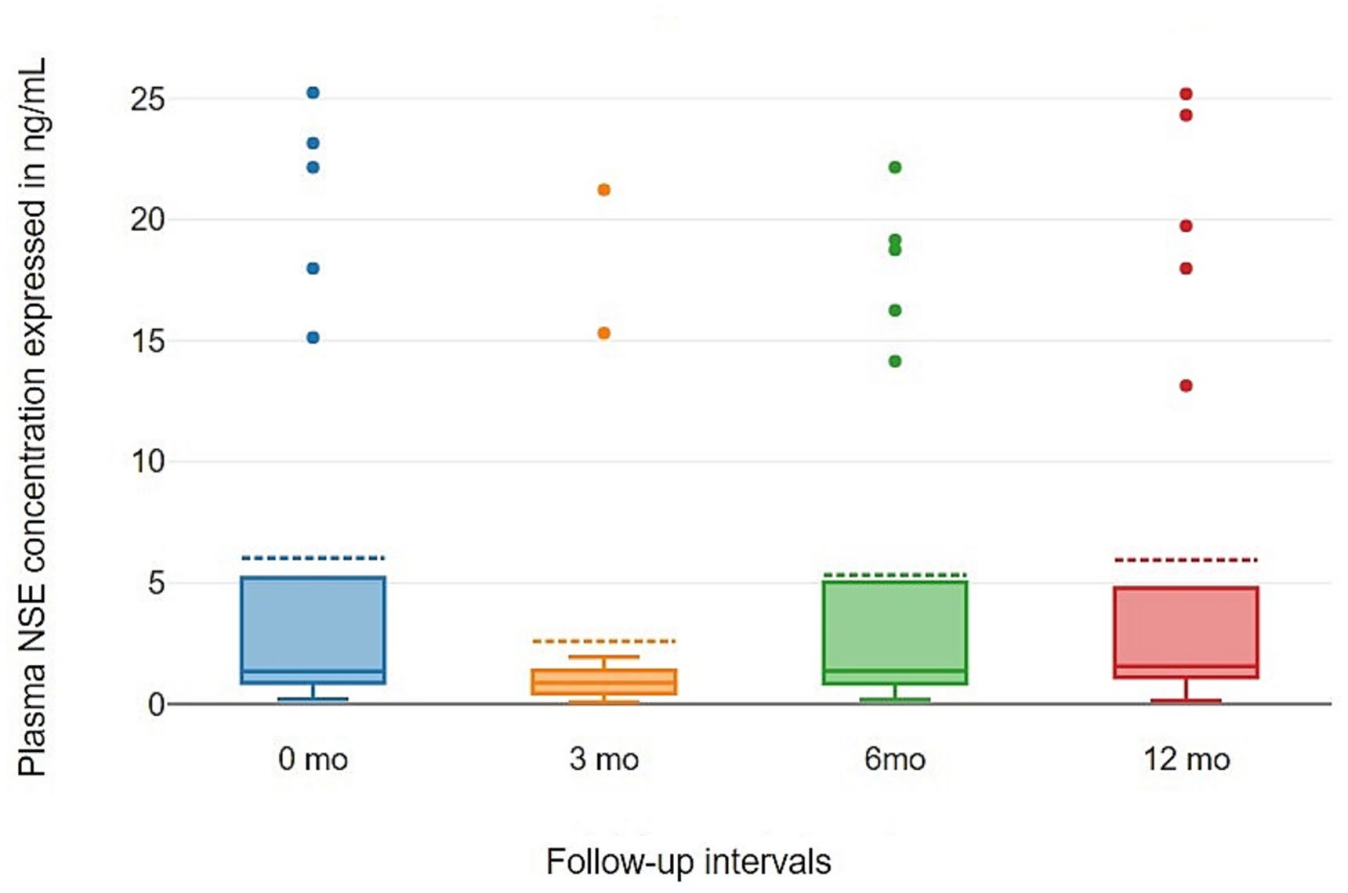
Visual analysis of the NSE distribution using box plots confirming the absence of a directional trend. The graph clearly reveals pronounced data scatter, with a subset of patients exhibiting NSE levels that exceeded the group median multiple-fold, reaching values as high as 25.24 ng/mL. Outliers with extreme NSE elevation were retrospectively reviewed; no acute neurological events (stroke, traumatic brain injury) were identified, suggesting these values reflect covert ictal activity.

The analysis of the longitudinal dynamics of neuron-specific enolase (NSE) during VNS therapy did not reveal statistically significant changes (Friedman test: *χ*^2^ = 5.34; d*f* = 3; *p* = 0.149). The baseline median NSE concentration was 1.34 [0.88; 5.20] ng/mL. By Month 12 of follow-up, the level remained stable at 1.56 [1.11; 4.78] ng/mL. As illustrated in Figure 7 NSE levels were characterized by extremely high inter-individual variability (minimum 0.08 ng/mL, maximum 25.24 ng/mL), which precluded the identification of a consistent trend in response to the treatment.

Consistently low median values across the 0, 3, 6 and 12-month time points, juxtaposed with such anomalous outliers, suggest that NSE levels reflect individual episodes of neuronal tissue injury rather than a systemic cumulative effect of VNS therapy.

Analysis of Neuron-Specific Enolase (NSE) dynamics revealed no statistically significant changes during therapy. The omnibus test (Friedman test) yielded a *p*-value of 0.149, exceeding the significance threshold of 0.05, which indicates the absence of a systemic impact of VNS on this biomarker.

Detailed post-hoc pairwise comparisons (Dunn-Bonferroni test) further failed to confirm significant dynamics: the adjusted *p*-value (Adj. *p*) for the majority of pairs was 1.0. Notably, even for the 3 months vs. 12 months interval, where the unadjusted value suggested a trend toward differentiation (*p* = 0.027), the result did not reach statistical significance following rigorous Bonferroni correction (Adj. *p* = 0.11). Simultaneously, extreme inter-individual variability was observed, characterized by isolated outliers reaching 25.24 ng/mL, reflecting the sporadic nature of marker elevation in specific patients.

Analysis of neuron-specific enolase (NSE) levels in the control group (*n* = 20) revealed no statistically significant changes over the 12-month follow-up period (Friedman test, *χ*^2^ = 0.77; d*f* = 3; *p* = 0.856). The median biomarker concentration remained stable across all control time points: 2.36 [1.80; 2.66] ng/mL at baseline, 2.35 [1.80; 2.66] ng/mL at 3 months, 2.34 [1.80; 2.66] ng/mL at 6 months, and 2.44 [1.80; 2.66] ng/mL by the end of the year.

**Figure 8:**
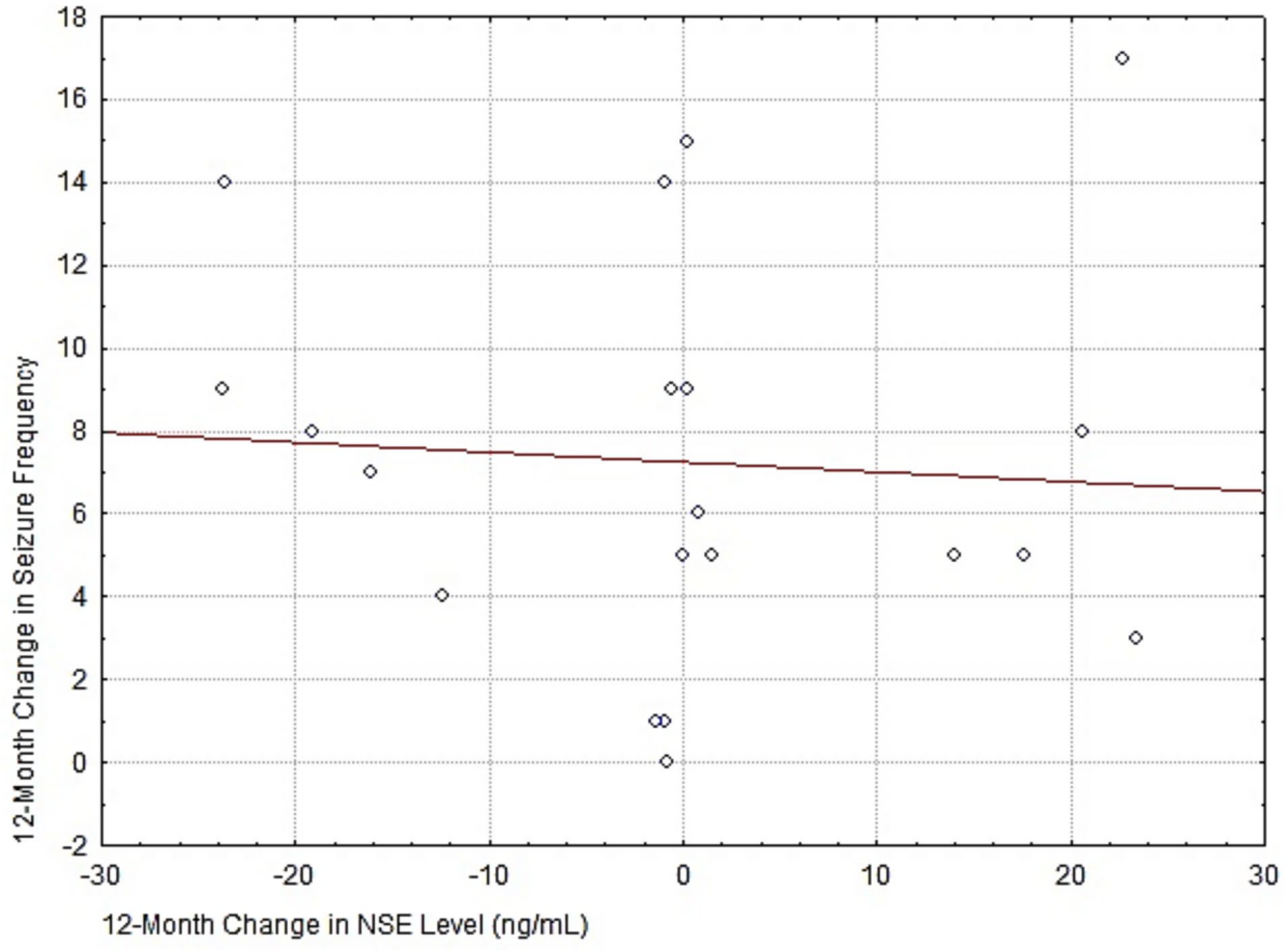
Spearman’s rank correlation coefficient was *r* = –0.04 (*p* > 0.05) indicating the absence of a clinically significant relationship.

The scatterplot in Figure 8 demonstrates the absence of a statistically significant relationship between the change in neuron-specific enolase levels and the reduction in seizure frequency by Month 12 of follow-up.

In Figure 8, Spearman’s rank correlation coefficient was *r* = −0.04 (*p* > 0.05) indicating the absence of a clinically significant relationship.

### 3.2 Clinical efficacy of VNS therapy

The primary clinical outcome was evaluated based on the reduction in seizure frequency over a 12-month follow-up period. In the active VNS therapy group, a progressive and statistically significant reduction in seizure frequency was observed. By month 12 of continuous neuromodulation, the median seizure frequency had decreased by 44.4% compared to baseline (*p* < 0.001). Conversely, the control cohort, receiving stable background anti-seizure medication without neuromodulation, did not exhibit significant changes in their clinical profile throughout the observation period (*p* > 0.05). This contrast confirms the specific therapeutic efficacy of VNS in this pediatric cohort.

### 3.3 Dynamics of neuroimmune biomarkers

Biomarker profiling in the active VNS group revealed different temporal dynamics for the investigated proteins. The plasma concentration of UCH-L1 demonstrated a significant decrease by month 6 of therapy (*p* = 0.009). Subsequently, the levels of HMGB1 exhibited a highly significant reduction at month 12 (*p* < 0.001). This decline in HMGB1 concentrations strongly correlated with the clinical reduction in seizure frequency (*r* = 0.63). In contrast, NSE levels did not show significant longitudinal alterations during the observation period. In the control group, the plasma levels of all investigated proteins (UCH-L1, HMGB1, and NSE) did not change significantly at any time point (all *p* > 0.05).

## 4 Discussion

This study presents a prospective longitudinal analysis of systemic molecular markers that potentially reflect the mechanisms underlying the clinical efficacy of VNS in children with drug-resistant structural epilepsy. Our findings align with the hypothesis that the therapeutic potential of VNS extends beyond the direct desynchronization of aberrant neural networks and may encompass profound and sustained neuroimmunomodulation. We demonstrated that clinical improvement—specifically, the reduction in seizure frequency—is strictly associated with temporal alterations in the profile of peripheral neuroimmune biomarkers.

Crucially, while the VNS intervention group exhibited a significant dynamic reduction in UCH-L1 and HMGB1 levels, the comparison group maintained a persistent plateau across all investigated markers throughout the 12-month follow-up period. This stark contrast suggests that optimal medical therapy alone is insufficient to mitigate systemic excitotoxic stress in this cohort, thereby underscoring the specific disease-modifying effect of neuromodulation.

The dynamics of UCH-L1 suggest the presence of an early phase of the therapeutic response to VNS. In our intervention cohort, a statistically significant decrease in UCH-L1 levels was observed as early as the 6th month. Given that UCH-L1 is a highly specific neuronal enzyme whose presence in the blood is strictly associated with the disruption of endothelial tight junctions, its early clearance may indirectly reflect the functional restoration of the blood–brain barrier (BBB). We hypothesize that this potential barrier stabilization is linked to the VNS-mediated suppression of local pro-inflammatory cytokines, which limits the pathological infiltration of the brain parenchyma by peripheral immune cells.

In contrast to the early decline in UCH-L1, a statistically significant drop in systemic HMGB1 levels was documented only by the 12-month follow-up. This delayed dynamic likely reflects a complex, cumulative process. Preclinical models demonstrate that VNS acts upon microglial α7-nicotinic acetylcholine receptors (α7nAChR), facilitating the physical degradation of TLR4 receptors. Because HMGB1 functions as a classic DAMP that binds to TLR4, we posit that the progressive systemic reduction of HMGB1 observed in our cohort reflects the attenuation of this inflammatory HMGB1-TLR4 loop.

An important finding was the lack of statistically significant longitudinal dynamics in systemic neuron-specific enolase (NSE) levels, despite a clear clinical response and the normalization of HMGB1 and UCH-L1. We interpret this phenomenon through the prism of systemic versus localized biomarker release. While UCH-L1 and HMGB1 trajectories appear to reflect widespread barrier stabilization and systemic anti-inflammatory loops, sporadic NSE fluctuations (characterized by extreme inter-individual variability) likely represent ongoing, localized subclinical epileptiform activity. Although this covert activity may be insufficient to generalize into a motor seizure, it is capable of inducing localized neuronal injury, provoking a continuous passive leakage of NSE into the bloodstream.

Thus, the persistence of peripheral NSE fluctuations in the setting of a clinical response does not necessarily indicate treatment failure, but rather reflects the ongoing clearance of protein debris from localized subclinical injury. It is plausible to speculate that this tissue sanitation process is synergistically supported by broader neuroprotective mechanisms. Specifically, the early normalization of UCH-L1—a critical enzyme for the ubiquitin-proteasome system (UPS)—suggests a potential restoration of intracellular protein homeostasis and the preservation of the intracellular enzyme pool required to clear aberrant aggregates. Furthermore, the overall reduction in systemic inflammation may theoretically facilitate enhanced glymphatic drainage, assisting in the continuous washout of neuronal degradation products (Plog and Nedergaard, 2018; Rasmussen et al., 2018; Pu et al., 2023). However, while systemic NSE is a less informative biomarker for monitoring the cumulative anti-inflammatory effects of VNS compared to UCH-L1 and HMGB1, the sustained reduction in inflammatory markers suggests that VNS reliably protects the surrounding intact parenchyma from the generalization of excitotoxic stress.

## 5 Conclusion

In the investigated pediatric cohort with drug-resistant structural epilepsy, VNS therapy was associated with marked clinical improvement. By the 12-month follow-up, a statistically significant reduction in median seizure frequency from 13.5 to 7.5 per month was achieved (*p* < 0.001), a finding consistent with data from large-scale international registries (Orosz et al., 2014; Englot et al., 2011). According to the McHugh classification, 40% of the cohort achieved a robust response (Class I–II), and 55% demonstrated a partial benefit (Class III). Consistent with data from large-scale international registries these findings confirm the cumulative nature of VNS therapy, demonstrating progressively enhanced therapeutic efficacy over time (Tan et al., 2025). Longitudinal analysis of the peripheral neuroimmune panel (UCH-L1, HMGB1, NSE) leads us to hypothesize that this clinical response may be accompanied by a phased systemic immunomodulation. The early decline in UCH-L1 levels by the 6th month may indirectly indicate the initial stabilization of the blood–brain barrier (BBB). The subsequent reduction in HMGB1 by the 12-month mark aligns with the premise of a delayed functional suppression of the DAMP-mediated inflammatory response. Notably, the lack of significant directional dynamics in NSE levels despite evident clinical improvement suggests that systemic NSE is less informative for monitoring the cumulative anti-inflammatory effects of VNS compared to the other evaluated biomarkers. Ultimately, while the observed biomarker trajectories are associative in nature, the longitudinal monitoring of this specific panel emerges as a promising translational tool. Following future validation in larger prospective cohorts, it could potentially serve as an objective adjunct for evaluating the individualized biological response to VNS, thereby facilitating the personalized optimization of stimulation parameters.

### 5.1 Study limitations

Despite the robust findings presented, this study is subject to several limitations and methodological considerations that warrant acknowledgment.

#### 5.1.1 Sample size and logistical constraints

The size of the interventional sample (*n* = 20) was dictated not only by stringent clinical inclusion criteria but also by the organizational and logistical frameworks governing the provision of high-tech medical care. In Kazakhstan, the implantation of vagus nerve stimulators is fully covered by state funding through a strict quota system. Under routine clinical practice conditions, our center’s operational capacity averages 10 implantations per year. To execute the present longitudinal design, we initiated a formal request to expand the quota, effectively doubling the annual surgical volume specifically for this study. Thus, the interventional cohort represents the maximum attainable sample size achieved through targeted administrative support.

#### 5.1.2 Etiological heterogeneity

The patients enrolled in the study presented with diverse structural etiologies of epilepsy (e.g., sequelae of hypoxic–ischemic injury, focal cortical dysplasias, and gliotic changes). Although all participants were classified as having “structural focal epilepsy,” the inherent differences in their pathomorphological substrates may have contributed to the variability observed in biomarker trajectories. Nevertheless, the longitudinal design, featuring assessments at four distinct time points for each patient, enables the robust tracking of intra-individual dynamics, thereby effectively mitigating the confounding influence of baseline etiological heterogeneity.

#### 5.1.3 Clinical monitoring and EEG limitations

The evaluation of seizure frequency relied predominantly on patient diaries and intermittent video-EEG monitoring. The absence of continuous 24-h EEG recordings throughout the entire 12-month follow-up period precludes the absolute exclusion of subclinical (non-convulsive) epileptiform activity. As discussed above, this factor may account for the persistence of elevated levels of certain markers (particularly NSE) even in patients with a significant reduction in visible motor seizures; covert epileptic discharges are capable of sustaining local neuronal stress and passive cytolysis in the absence of overt motor manifestations.

A recognized limitation of the present study is the exclusive use of peripheral blood for biomarker quantification. Although evaluating circulating proteins restricts our ability to draw definitive conclusions regarding localized neuroinflammatory processes within the central nervous system, the observed delayed attenuation of systemic inflammatory markers remains highly informative. This systemic anti-inflammatory shift represents a critical component of the cumulative clinical benefits provided by VNS. Consequently, despite this anatomical disconnect, the longitudinal tracking of this specific neuroimmune panel offers a practical, minimally invasive surrogate marker of therapeutic efficacy. In clinical practice, such accessible profiling could ultimately facilitate the individualized optimization of neurostimulation settings.

## Data Availability

The datasets presented in this study can be found in online repositories. The names of the repository/repositories and accession number(s) can be found in the article/supplementary material.
